# Impact of mode of delivery on pregnancy outcomes in women with premature rupture of membranes after 28 weeks of gestation in a low-resource setting: A prospective cohort study

**DOI:** 10.1371/journal.pone.0190388

**Published:** 2018-01-10

**Authors:** Herbert Kayiga, Felicia Lester, Pauline Mary Amuge, Josaphat Byamugisha, Amy Meg Autry

**Affiliations:** 1 Directorate of Obstetrics and Gynecology, Makerere University College of Health Sciences, Kampala- Uganda; 2 Department of Obstetrics and Gynecology& RS, University of California San Francisco, San Francisco, California, United States of America; 3 BAYLOR College of Medicine, Kampala-Uganda; UH Case Medical Center, UNITED STATES

## Abstract

**Background:**

Despite the high prevalence of premature rupture of membranes (PROM) in low-resource settings, the preferred mode of delivery remains unclear. We compared the perinatal mortality in a prospective cohort of women with PROM after 28 weeks following vaginal or caesarean delivery at Mulago Hospital with the aim of adopting evidence based practice and improving patient care.

**Methods:**

Between November 2015 and May 2016, 1455 women with PROM after 28 weeks of gestation and their newborns were prospectively followed from admission to discharge at Mulago Hospital. The primary outcome was perinatal mortality. Secondary neonatal outcomes included sepsis and admission to the Special Care Unit. Maternal outcomes included maternal deaths and complications. Outcomes were compared between women who had vaginal vs. caesarean delivery using multivariable logistic regression. All statistical tests were 2-sided with the level of statistical significance set at p < 0.05.

**Results:**

The incidence of PROM was 12.1%. The perinatal mortality following PROM was 65 per 1000 live births. Of the 1425 women with PROM, 991 (69.5%) had vaginal delivery and 434 (30.5%) underwent Caesarean section. There was no statistical difference in perinatal mortality by the mode of delivery (vaginal vs. caesarean) in PROM (p = 0.12). The risk factors for perinatal mortality included chorioamnionitis, failure to administer corticosteroids in preterm PROM, gestational age (28–33 weeks), duration of drainage of liquor (24–48 hours), and presence of maternal complications. Caesarean delivery was associated with increased maternal postpartum infections, admission to the Special Care Unit and maternal death.

**Conclusion:**

In low resource settings, vaginal delivery is the preferred mode of delivery for PROM after 28 weeks gestation. It is associated with lesser maternal and perinatal morbidity when compared to caesarean delivery.

## Introduction

Premature rupture of membranes (PROM) an obstetric condition that involves spontaneous amniotic fluid leakage at least one hour before onset of labor, complicates 5–10% of all deliveries[1]. PROM contributes to adverse maternal morbidity and mortality including chorioamnionitis, endomyometritis, postpartum hemorrhage, pelvic abscess, and increased likelihood of caesarean delivery[2]. Preterm PROM is associated with increased perinatal morbidity and mortality, especially when it occurs remote from term [3].

Although caesarean delivery is often recommended, there is no consensus on the ideal delivery mode in PROM in resource limited settings [4–8]. Management of PROM is particularly challenging in low-resource settings, where access to caesarean delivery may be limited and may result in long waiting times for surgery. More vaginal deliveries could potentially help decrease time to delivery and thus reduce the risk of maternal infections, stillbirths and neonatal morbidity and mortality. If providers had evidence that vaginal birth was as safe as or safer than caesarean delivery in low-resource settings, they could feel comfortable inducing women with PROM.

As there is minimal neonatal survival in Uganda before 28 weeks of gestation, we compared the maternal and perinatal outcomes of vaginal to caesarean delivery at Mulago Hospital in a prospective cohort of women with PROM after 28 weeks of gestation.

## Materials and methods

### Study design

We conducted a prospective cohort study of 1455 women with premature rupture of membranes after 28 weeks of gestation between November 2015 and May 2016 at Mulago Hospital. Eligible participants were followed from admission to discharge. Twenty-eight weeks was chosen for entry criteria, as this is the accepted gestational age for viability in Uganda.

### Study setting

This study was conducted in the obstetric wards of Mulago Hospital in Kampala, Uganda. Mulago Hospital is the national referral hospital for Uganda and also the teaching hospital for Makerere University College of Health Sciences. It is a government-funded hospital with a bed capacity of 1700, with approximately 300 beds allocated to obstetrics and gynecology. The study units included the labor suite, postnatal ward, labor suite operating theatre, high observation unit, and the Neonatal Special Care Unit. The units operate 24 hours per day and offer free services to the public. Patients come from within Kampala, as well as neighboring and distant districts.

At Mulago hospital, 20–25 caesarean deliveries are done in a 24-hour shift. Despite this volume of caesarean deliveries, at any one time, there are 10–30 pending caesarean sections. Because of this long theatre queue, a number of expectant mothers scheduled to have caesarean delivery don’t access this intended delivery on time. This quite often leads to adverse pregnancy outcomes at the facility.

### Participants

Pregnant women admitted at Mulago Hospital who had premature rupture of membranes after 28 weeks of gestation and at least one hour before onset of spontaneous labor with viable singleton pregnancies were included. Those with multiple gestations, babies with known congenital anomalies, and intrauterine fetal deaths were excluded. Pregnant women who did not report history of drainage of liquor or who did not have leakage of liquor on examination on admission were also excluded.

#### Categorization of the participants

Participants were categorized either as preterm PROM (gestation age between 28–36 weeks and 6 days) or term PROM (≥37 weeks but before onset of labor). Gestational age was determined using the participants’ first day of the last normal menstrual period or by any available antenatal obstetric ultrasound scan reports. First trimester ultrasound scans were preferred, though in their absence any available ultrasound scan reports were used.

### Diagnosis of PROM

A diagnosis of PROM was made when spontaneous membrane leakage was reported at least one hour before onset of labor. In addition to a routine obstetric examination, a speculum examination (when speculums were available) was used to confirm pooling of liquor in the vagina. Mulago Hospital does not offer free ultrasound services. When patients paid, an obstetric ultrasound was performed to determine viability, liquor pool, gestational age, presentation or presence of any obvious fetal anomalies.

Trained staff using pretested data extraction tools, collected participants’ socio-demographic characteristics, neonatal and maternal variables as stated below.

#### Management options

Patient management was expectant, active (induction of labor with either Misoprostol or Pitocin infusion), or by caesarean delivery. Expectant management in this study was when participants were given conservative treatment after a diagnosis of PROM was made but with planned vaginal delivery. Expectant management was implemented for women with PROM remote from term, and those who were on corticosteroids for fetal lung maturity. Induction of labor was indicated for participants with PROM from 34 weeks with no contraindication to vaginal delivery. Caesarean delivery included participants scheduled for elective caesarean delivery without a trial of labor or emergency caesarean when the baby’s or maternal condition was in jeopardy. The indications for caesarean section in participants with PROM included, remoteness from term (28–32 weeks), fetal mal-presentation, severe oligohydramnios, prior caesarean scars especially when more than one scar, inability to do optimal fetal surveillance, chorioamnionitis and presence of non-reassuring fetal heart.

### Outcome variables

#### Primary outcome

Perinatal death defined as fresh stillbirth or neonatal death within the first seven days of life.

#### Secondary outcomes

**S**econdary neonatal outcomes included sepsis(defined as presence of a fever with any of the following; convulsions, failure to breastfeed, difficulty in breathing, cough, vomiting, diarrhea or any obvious skin infection) and admission to the neonatal Special Care Unit (decision for admission followed either use of locally available protocols or clinician’s judgment). Secondary maternal outcomes included death and maternal complications including chorioamnionitis (defined as having any of the following; temperature of 38°C or higher, uterine tenderness and foul smelling discharge), postpartum hemorrhage; wound infection, or wound separation.

### Co-variables

To characterize the population and allow adjustment for potential confounding, we collected information on parity, maternal age, duration of rupture of membranes, method of induction, decision to incision time for caesarean delivery, administration of antibiotics or corticosteroids, characteristics of fetal monitoring, and HIV sero-status.

### Ethical consideration

IRB approvals were obtained from the University of California San Francisco, and Mulago Hospital Institutional review boards, the Directorate of Obstetrics and Gynecology, Makerere University, and written informed consent was obtained from all study participants.

### Statistical analysis

Data entry was performed with EPI-DATA 3.1 and analyzed using STATA version 12. Baseline characteristics were compared using descriptive statistics presented in frequencies, means and percentages as appropriate. We used multilevel logistic regression to determine the impact of mode of delivery on perinatal mortality. Variables in Table 1 that were associated with perinatal mortality with p-value ≤ 0.2 in Univariate analysis were entered into a multivariate logistic model with mode of delivery. Interaction among variables in the multivariate model was checked. At sample size calculation, power of 80% was used. However, the post-analysis power was 87%.

**Table 1:** Baseline characteristics of 1425 participants with PROM at Mulago Hospital, Kampala.

Variable	Vaginal Delivery (n = 991) n(%)	Caesarean Section (n = 434) n(%)	p-value
**Age**			
< 18 years	30 (3.0)	8 (1.8)	0.17
18–24	511 (51.6)	205 (47.2)	
25–35	401 (40.5)	199 (45.9)	
> 35 years	49 (4.9)	22 (5.1)	
**Current smoker**			
Yes	18 (1.8)	8 (1.8)	
No	973 (98.2)	426 (98.2)	0.95
**Parity**			
Prima gravida	415 (41.9)	154 (35.5)	**0.02**
Para 2–3	343 (36.4)	184 (42.4)	
Para ≥ 4	233(23.5)	96 (22.1)	
**HIV Status**			
Positive	112 (11.3)	56 (12.9)	0.66
Negative	879 (88.7)	378 (87.1)	
**Fever**			
Yes	52 (5.2)	32 (7.4)	0.11
No	939 (94.8)	402 (92.6)	
**Referral status to Mulago hospital**			
Referred	717 (72.4)	298 (68.7)	0.17
Not referred	274 (27.6)	136 (31.3)	
**Gestational age**			
28–33 weeks	110 (11.1)	60 (13.8)	**0.01**
34–36 weeks	278 (28.1)	89 (20.5)	
≥37 weeks	603 (60.8)	285 (65.7)	
**Duration of drainage**			
<24 hours	437 (44.1)	187 (43.1)	0.80
24–48 hours	321 (32.4)	140 (32.3)	
2–7days	206 (20.8)	89 (20.5)	
>7days	26 (2.6)	18 (4.1)	
**Color of liquor**			
Clear	884 (89.2)	374 (86.2)	0.07
Light meconium	99 (10.0)	51 (11.8)	
Thick meconium	8 (0.8)	9 (2.0)	
**Smell of liquor**			
Offensive	63 (6.4)	51 (11.8)	**<0.01**
Not offensive	928 (93.6)	383 (88.2)	
**Speculum exam done**			
Yes	27 (2.7)	18 (4.1)	0.15
No	964 (97.3)	416 (95.9)	
**Digital exams per day**			
None	22 (2.2)	14 (3.2)	0.67
1–2	523 (52.8)	224 (51.6)	
3–4	344 (34.7)	154 (35.5)	
≥5	102 (10.3)	42 (9.7)	
**Fetal Heart Present**			
Yes	984 (99.3)	434 (100.0)	0.08
No	7 (0.7)	0 (0.0)	
**Obstetric US at time of admission**			
Yes	647 (65.3)	207 (47.7)	**<0.01**
No	344 (34.7)	227 (52.3)	

p-values <0.05 were significant

## Results

### Baseline characteristics of participants

From November 2015 to May 2016, 12,022 women delivered 12,377 babies at Mulago Hospital. The incidence of PROM during this period was 12.1%. We recruited 1455 eligible participants; 30 were lost to follow-up and analysis included data for 1425 participants, of whom 888 (62.3%) had term PROM. Of the 1425 women with PROM, 991 (69.5%) had vaginal delivery and 434 (30.5%) underwent caesarean section (87 participants had elective while 347 had emergency caesarean delivery). The mean age of the participants was 25 years (SD±5.3), 40% were prima gravida, mean parity was 2, the mean gestational age was 36 weeks, and only 26 participants (2%) reported cigarette smoking (Table 1).

From the baseline and obstetric characteristics, the following variables were independently associated with the mode of delivery: gestational age (p-value = 0.009), smell of liquor (p-value <0.001), an obstetric scan done (p-value <0.001), history of drainage of liquor (p-value = 0.05) and parity (p-value = 0.02) (Table 1).

### Effect of mode of delivery on maternal outcomes

Caesarean delivery was associated with both maternal morbidity and mortality. There was one maternal death following caesarean delivery while none followed vaginal delivery. All infection-related postpartum morbidities occurred after caesarean, rather than vaginal delivery. These infection-related morbidities included seven participants with puerperal sepsis and one with pelvic abscess (Table 2).

**Table 2:** Comparison of maternal and neonatal outcomes between vaginal and caesarean section at Mulago Hospital.

Variable	Vaginal Delivery (n = 991) n(%)	Caesarean Section(n = 434) n(%)	p-value
**Neonatal Outcome Variables**			
**APGAR score (1 min)**			
0–3	53 (5.31)	24 (5.5)	0.81
4–6	97 (9.7)	48 (11.1)	
7–10	841 (85)	362 (83.4)	
**APGAR score (5 min)**			
0–3	36 (3.6)	17 (3.9)	0.86
4–6	27 (2.7)	10 (2.3)	
7–10	928 (93.6)	407 (93.8)	
**Baby’s Outcome**			
Alive	929 (93.7)	398 (91.7)	0.15
Died	62 (6.3)	36 (8.2)	
**Baby admitted to SCU**			
Yes	201 (20.3)	123 (28.3)	**0.01**
No	790 (79.7)	311 (71.6)	
**Maternal Outcome Variables**			
**Maternal complication**			
Chorioamnionitis	34 (3.4)	35 (8.1)	**0.02**
Pelvic Abscess	0 (0.0)	1 (0.2)	
Puerperal sepsis	0 (0.0)	7 (1.61)	
PPH	19 (1.8)	8 (1.8)	

p-values <0.05 were significant

#### Maternal HIV status

There were 168 (11%) HIV sero-positive participants in this study. HIV sero-status did not have a statistically significant impact on maternal or fetal outcome (p = 0.68) even at multivariate analysis.

### Effect of mode of delivery on neonatal outcomes

#### Admission to Special Care Unit (SCU)

**Fig 1:**
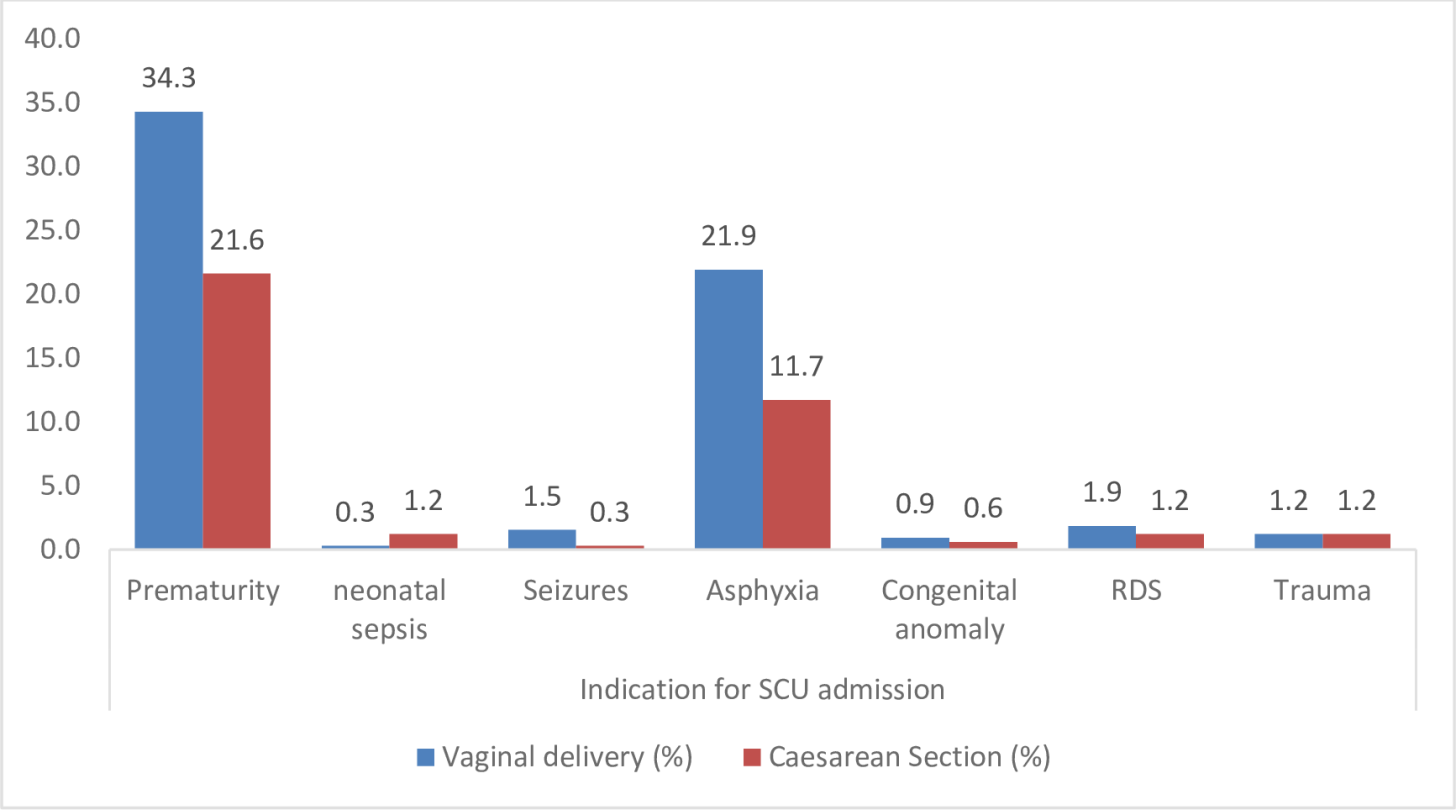
Fetal conditions leading to the 324 Special Care Unit admissions as per the different modes of delivery at Mulago Hospital. Fig 1 shows that Prematurity was the leading condition for the admission followed by asphyxia. Congenital anomalies contributed the least to the admission. RDS is Respiratory Distress Syndrome.

Prematurity was the commonest indication for admission into the Special care Unit (181/324 cases, 56%) as shown in “Fig 1”.

There was no statistically significant relationship between HIV status of the mother and the admission to SCU or neonatal sepsis. The odds ratio of admission to SCU was higher among babies born by caesarean delivery, compared to those delivered vaginally: 1.57 times higher (95% CI 1.21–2.03, p-value = 0.001). The odds ratio of perinatal death was 3.71 times higher (95% CI 2.24–5.68, p-value <0.001) among babies admitted to SCU, compared to babies not admitted.

### APGAR score

Apgar score rating scale evaluation showed no significant difference in the different modes of delivery (p = 0.81) as shown in Table 2.

### Risk factors for perinatal death

#### Consistency and smell of liquor

Compared to those with clear liquor, the odds ratio of perinatal mortality following light meconium stained liquor was 2.24 (95% CI 1.29–3.87; p-value = 0.004). This was higher 3.52 (1.00–12.52; p-value = 0.05) among those with thick meconium.

The smell of liquor had also a statistically significant impact on perinatal mortality. For those with an offensive meconium odor, the odds ratio of perinatal death was 2.20 (95% CI 1.20–4.02, p-value = 0.011) as compared to those with non-offensive liquor.

#### Presence of maternal complications and adverse neonatal perinatal outcomes

The risk of perinatal mortality was higher when maternal complications occurred, including: chorioamnionitis, puerperal sepsis, pelvic abscess, postpartum hemorrhage and maternal death (Table 3). The odds ratio of perinatal death was 3.81 (95% CI: 2.20–6.61, p-value <0.001) when any one of the maternal complications occurred.

**Table 3:** Maternal and neonatal characteristics associated with perinatal death.

Variable	Baby alive (n = 1332) n(%)	Perinatal death (n = 93) n(%)	p-value
**Planned mode of delivery**			
Expectant	655 (49.2)	47 (50.5)	0.80
Induction of labor	337 (25.3)	15 (16.2)	
Caesarean section	340 (25.5)	31 (33.3)	
**Actual mode of delivery**			
Vaginal	933 (70.0)	58 (62.4)	0.12
Caesarean Section	399 (30.0)	35 (37.6)	
**Birth weight**			
<2.5 kg	275 (20.6)	44 (47.3)	**<0.01**
2.5–3.5 kg	831 (62.4)	39 (41.9)	
>3.5 kg	216 (16.2)	10 (10.8)	
**Gestational age**			
28–33 weeks	137 (10.3)	33 (35.5)	**<0.01**
34–36 weeks	348 (26.1)	19 (20.4)	
≥ 37 weeks	847 (63.6)	41 (44.1)	
**History of drainage of liquor**			
Yes	270 (20.3)	20 (21.5)	0.78
No	1062 (79.7)	73 (78.5)	
**Corticosteroids given for gestation age <34 weeks**			
Yes	175 (87.1)	30 (76.9)	0.10
No	26 (12.9)	9 (20.1)	
**Antibiotics given**			
Yes	921 (69.1)	63 (67.7)	0.79
No	411 (30.9)	30 (33.3)	
**Indication for Antibiotic**			
Prophylaxis	865 (95.8)	46 (73.0)	**<0.01**
Treatment	56 (4.2)	17 (27.0)	
**Overall length of hospital stay for the mother**			
1 day	40 (3.0)	5 (5.4)	0.44
2–3 days	737 (55.3)	39 (41.9)	
≥ 4 days	555 (41.7)	49 (52.7)	
**Maternal complication**			
Chorioamnionitis	52 (61.9)	17 (89.6)	0.08
Pelvic Abscess	1 (1.2)	0 (0.0)	
Puerperal sepsis	7(7.1)	1 (5.2)	
PPH	25 (29.8)	1 (5.2)	
**Baby admitted to SCU**			
Yes	278 (20.9)	46 (49.5)	**<0.01**
No	1054 (79.1)	47 (50.5)	
**Duration of admission to SCU**			
< 24 hours	12 (4.3)	16 (34.8)	**<0.01**
24–72 hours	92 (33.1)	22 (47.8)	
>72 hours	174 (62.6)	08 (17.4)	

p-values <0.05 were significant

Chorioamnionitis was the commonest maternal complication in the study (69/104 cases, 66%: see Table 3). Of the 69 participants with chorioamnionitis, 65 (94.2%) had more than 1 digital exam per day. Only 45 (3%) of the participants had speculum examination on admission. Nearly half of the participants had more than three vaginal examinations in 24 hours (640 cases, 45%).

#### Effect of antibiotic administration on perinatal mortality

Antibiotic use did not have any significant influence on perinatal mortality (p-value = 0.79); nor did it have an influence on the occurrence of neonatal complications like sepsis (p-value = 0.88). The maternal indication for the antibiotic use especially when it was given for chorioamnionitis significantly influenced the perinatal mortality. The odds ratio of perinatal death was 5.70 (95% CI: 3.07–10.59, p-value <0.001) for mothers who got antibiotics as treatment rather than prophylaxis for chorioamnionitis.

#### Impact of corticosteroid administration on perinatal outcome

Of the 240 participants who were eligible for corticosteroid administration (gestation age < 34 weeks), 201 (83.7%) received the prophylactic doses. Corticosteroid use significantly improved the perinatal outcomes following preterm PROM (Table 3).Preterm babies who did not receive corticosteroids intrauterine had 2.22 times higher odds ratio of perinatal death (95% CI: 1.44–3.45, p-value <0.001) compared to those that had intrauterine exposure to corticosteroids. This was more significant among preterm babies remote from term.

#### Gestational age and birth weight

Gestational age had a statistically significant impact on the perinatal outcome (Table 4).The perinatal mortality of term PROM was 46 per 1000 live births while that of preterm PROM was 97 per 1000 live births. The odds ratio of babies with birth weight <2.5kg dying in the perinatal period was 3.34 (95% CI 2.11–5.28, p-value <0.001).

**Table 4:** Multivariate analysis showing risk of perinatal death.

Variable	OR (95% CI)	p-value
Duration of drainage of liquor (24–48 hours)	1.81 (1.05–3.12)	0.031
Occurrence of complication for mother	5.10 (2.78–9.37)	<0.001
Gestation age 28–33 week	3.70 (2.09–6.55)	<0.001
Mode of delivery (Caesarean Section)	1.19 (0.75–1.89)	0.45

p-values <0.05 were significant

### Multivariate analysis

#### Effect of mode of delivery on perinatal death

There were 93 perinatal deaths (mortality rate of 65 per 1000 live births), of which 60 (64.5%) were early neonatal deaths. The crude rate of perinatal mortality was 5.9% with vaginal delivery vs. 8.1% with Caesarean delivery. At multivariable analysis, there was no statistically significant impact on perinatal mortality as per the different modes of delivery (OR1.19; 95% CI: 0.75–1.89, p-value 0.45). Presence of a maternal complication, (OR 5.10, 95% CI: 2.78–9.37, p < 0.001), earlier gestation age (between 28 and 33 weeks vs.>37weeks) (OR 3.70, 95% CI: 2.09–6.55, p <0.001) and longer duration of drainage of liquor (24–48 hours vs. < 24hours) (OR 1.81, 95% CI: 1.05–3.12, p-value 0.031) were independent risk factors associated with perinatal mortality (Table 4).

## Discussion

In this study, premature rupture of membranes had an incidence of 12.1%. The perinatal mortality rate in our study was 65 per 1000 live births. The mode of delivery did not have a statistically significant impact on perinatal mortality. Caesarean delivery was associated with more adverse pregnancy outcomes compared to vaginal route of delivery. The main risk factors for perinatal mortality included gestational age (28–33 weeks), failure to administer corticosteroids, drainage of liquor for more than 24 hours, presence of maternal complications, foul smelling and meconium stained liquor, and admission of the babies to neonatal Special Care Unit.

Our study’s incidence of PROM is higher than that reported by Caughey (5–10%)[1], Snehamay(2–4%)[9] and Revathi (7.86%)[10]. The higher incidence could have resulted from the fact that there is a high turnover of deliveries at the Mulago Hospital (33,000 deliveries/ annum). There could also have been over-documentation of PROM by the clinical team.

Despite the results reported by Chakraborty[4]and Mousiolis et al[5], recommending elective caeserean delivery in PROM so as to improve neonatal perinatal outcomes, in our study, mode of delivery did not significantly impact perinatal mortality.

In our study, vaginal delivery for PROM patients was found to be safer than caesarean delivery. The odds ratio of perinatal mortality for caesarean section was 1.19 higher than vaginal delivery. The odds ratio of neonatal admission to SCU was 1.57 higher for caesarean compared to vaginal delivery. The only maternal death in the study followed caesarean delivery. Kyung et al [11]also reported that postpartum infections were more common after caesarean delivery.

Thirty percent of the deliveries in our study were caesarean. The proportion of caesarean delivery was higher than that reported by other researchers. Tahir et al had a caesarean section proportion in PROM of 14%[6]. Eleje reported a caesarean section proportion of 23% while Ibishi et al reported one of 28%[7, 8]. Other studies however had higher cesarean proportions: Tavassoli et al reported a caesarean section proportion in PROM of 32%[12], while Pasquier et al reported a caesarean section rate of 58.7%[13].

In our study, 69.5% of the women with PROM had vaginal delivery which is lower than the 81.1% reported by Eleje in Nigeria [7], and 86% by Tahir et al [6]. The lower percentage of vaginal deliveries could have been attributed to the notion at the study site that caesarean delivery had better outcomes than vaginal delivery and the lack of consensus on best mode of delivery.

The perinatal mortality rate in our study of 65 per 1000 live births was lower than that reported by other researchers: 105–190 per 1000 live births [6, 14, 15]. The lower perinatal mortality in our study could have resulted from the fact that neonates were only followed from admission to discharge. Neonatal death could have occurred after discharge. This perinatal mortality rate is however higher than the Uganda national perinatal mortality rate of 40 per 1000 live births [16].

The incidence of chorioamnionitis in our study was 4.8%, accounting for 66% of all maternal complications. This incidence is higher than that reported by other researchers: 0.78–2.5% [11, 17, 18]. The relatively high prevalence of chorioamnionitis in our study could be attributed to multiple digital examinations by the clinical team. Only three percent of the participants had speculum examinations. Sterile speculums are frequently unavailable due to stock outs at Mulago hospital and providers rely on digital exams to assess labor.

Just as we found presence of chorioamnionitis to be associated with higher neonatal perinatal mortality, Kyung et al also reported an increased perinatal mortality following chorioamnionitis [11].

The odds of perinatal mortality in this study were higher when corticosteroids were not administered. This effect was more pronounced in earlier gestational ages remote from term, where odds ratio of perinatal mortality was 2.22 higher when corticosteroids were not administered compared to neonates of same gestational age range who got the steroids. Other researchers have also reported improved perinatal outcomes after corticosteroid administration [19–21]in PROM before 36 weeks.

We also observed that the odds of perinatal mortality were 3.7 folds higher when drainage of liquor was relatively longer than 24 hours. This finding has also been observed in other studies [11].

The strength of this study is that we were able to follow 98% of all our participants from admission to discharge. We were able to recruit 1455 participants, a larger cohort than prior studies in PROM. This study is conducted in a low resource setting and reflects the actual experience of poor access to basic equipment and problems with the timely ability to perform intended cesareans due to staffing and/or sheer volume.

The limitations of this study included the fact that some of the participants planned for caesarean delivery ended up delivering vaginally before they could access the operating rooms. Quite often, such patients ended up having poorer outcomes than when the initial planned mode of delivery had been vaginal. While it is standard of care to perform a speculum examination to diagnose PROM, the large patient load and institutional financial constraints prohibited this examination in many patients.

We acknowledge that relying mostly on patients’ history and clinical examination to confirm the diagnosis of PROM may lead to both an under and over inclusion of subjects into our study. It is difficult to know if one would be favored over another.

## Conclusion

Though there was no statistically significant difference in perinatal neonatal mortality according to our study as per the different modes of delivery, in low-resource settings, vaginal delivery is a safer mode of delivery. Vaginal delivery is associated with less maternal and perinatal morbidity as compared to caesarean. This data supports the practice that has been adopted in high-income countries of planned vaginal delivery for PROM and should be adopted as the standard of practice in low and middle-income countries.

## Abbreviations

HIV: Human Immunodeficiency Virus
IRB: Institutional Review Board
PROM: Premature Rupture of Membranes
PPROM: Preterm Premature Rupture of Membranes
SCU: Neonatal Special Care Unit
